# The Influence of Fatty Acid Expression Methods on Associations with Cardiometabolic Biomarkers: Evidence from NHANES 2011–2012

**DOI:** 10.3390/nu18172905

**Published:** 2026-09-04

**Authors:** Brian Hallmark, Manja M. Zec, Laurel Johnstone, Carrie S. Standage-Beier, Susan Sergeant, Justin M. Snider, J. Thomas Brenna, Floyd H. Chilton

**Affiliations:** 1 BIO5 Institute, University of Arizona, Tucson, AZ 85721, USA; bhallmar@arizona.edu; 2 Department of Orthopedics, University of Colorado Anschutz Medical Campus, Aurora, CO 80045, USA; manja.zec@cuanschutz.edu; 3 School of Nutritional Sciences and Wellness, College of Agriculture Life and Environmental Sciences, University of Arizona, Tucson, AZ 85721, USA; laureljo@arizona.edu (L.J.); cstandagebeier@arizona.edu (C.S.S.-B.); justinsnider@arizona.edu (J.M.S.); 4 Department of Biochemistry, Wake Forest School of Medicine, Winston-Salem, NC 27157, USA; susan.sergeant@wfusm.edu; 5 Dell Pediatric Research Institute, University of Texas at Austin, Austin, TX 78723, USA; tbrenna@utexas.edu; 6 Department of Pediatrics, University of Texas at Austin, Austin, TX 78723, USA; 7 Department of Chemistry, University of Texas at Austin, Austin, TX 78723, USA; 8 Department of Nutrition, University of Texas at Austin, Austin, TX 78723, USA; 9 Center for Precision Nutrition and Wellness, University of Arizona, Tucson, AZ 85721, USA; 10 Escuela de Medicina y Ciencias de la Salud, Tecnologico de Monterrey, Monterrey 64710, Mexico

**Keywords:** lipid biology, linoleic acid, fat Recommendations, fatty acids, PUFAs

## Abstract

**Background/Objectives:** Fatty acids (FAs) are critical mediators of human physiology, yet associations between dietary intake, circulating FA levels, and health outcomes remain debated. Circulating FAs are typically reported as a percentage of total FA (% total), whereas absolute concentrations are measured infrequently. Although mathematically related, these metrics provide distinct information. NHANES 2011–2012 uniquely provided serum FA concentrations, enabling direct comparison of concentration and % total within the same individuals. **Methods:** Serum FAs measured by electron capture negative-ion mass spectrometry in NHANES 2011–2012 were expressed as both absolute concentrations (µmol/L) and % total. Associations of each representation with lipid and non-lipid cardiometabolic biomarkers were evaluated and compared with total FA concentrations. **Results:** For multiple FAs and biomarkers, the direction of association differed depending on whether FAs were expressed as concentration or % total. These reversals occurred when total FAs varied more strongly with the biomarker than the individual FA concentration, allowing a FA to increase in absolute concentration while decreasing as a percentage of the total FA pool. Reversals were observed for several major FAs, including linoleic acid (LA), arachidonic acid (ARA), docosapentaenoic acid (DPA), docosahexaenoic acid (DHA), and stearic acid (SA). Total FA concentrations were strongly associated with most biomarkers. **Conclusions**: FA concentrations and % total are complementary, not interchangeable, measures that capture distinct aspects of circulating FA biology. Associations with outcomes can be in opposite directions when the total plasma FA pool varies. These differences reflect underlying mathematical and biological relationships rather than methodological error. Future studies should routinely evaluate the total FA pool size alongside FA composition. Examining both representations alongside total FA can help distinguish changes in absolute FA abundance from changes in relative composition and provide a more complete interpretation of FA associations.

## 1. Introduction

Fatty acids (FA) play essential roles in many biological processes, and researchers have sought for decades to understand the relationships between dietary and circulating FA levels and health outcomes. Levels of saturated FAs, polyunsaturated fatty acids (PUFA), and highly unsaturated fatty acids (HUFA) have been associated with multiple biomarkers and diseases, including cardiovascular disease (CVD), diabetes, and mortality [1,2,3,4,5]. Recent cohort analyses, pooled biomarker studies, and reviews continue to support the relevance of circulating FAs for cardiometabolic risk, while also highlighting ongoing uncertainty regarding the biological and clinical interpretation of specific FAs such as linoleic acid (LA), arachidonic acid (ARA), and eicosapentaenoic acid (EPA) [2,3,6,7,8,9,10,11]. Since the 1960s, FA quantitation data have primarily been generated by gas chromatography flame ionization detection (GC-FID) either in all circulating lipid classes or phospholipids. These data are typically presented as a percentage of the total FA pool being measured (% total), and corresponding absolute FA concentrations are not typically provided [1,12,13,14,15,16,17,18,19,20]. This is partially because peak-based chromatography data are naturally compositional (the areas under the individual peaks sum to the total area), and obtaining concentrations requires an additional step of using internal standards to convert areas to concentrations, which can introduce error from creating and using standard curves. Consequently, much of the epidemiologic evidence linking circulating FAs to cardiometabolic outcomes has relied on FA values expressed as relative proportions rather than absolute circulating concentrations (for example, [2] examines 49 studies that all use % total).

This use of % total makes the data compositional, as the proportions of the FA in the total FA pool must sum to one. Due to this sum constraint, if one FA changes, the level of one or more other FAs must change in the opposite direction. Using absolute concentrations avoids this, as individual FA values can vary independently from one another. However, it is often implicitly assumed that % total data can be treated like concentration data in that single FAs can be treated independently, and the overall association patterns would be the same as using concentrations. This is correct only when the total FA pool is not changing, an unlikely condition that is rarely checked. When the total FA pool is changing, Sergeant et al. demonstrated that associations between lipid-based clinical biomarkers with PUFAs and HUFAs, such as LA and ARA, often change direction when comparing % total values with concentrations [21]. This group presented a mathematical framework that determined the conditions for when associations reverse and showed the phenomenon empirically in two small data sets. This is based on the mathematical relationship: FA% = FA conc/Total FA. If the denominator changes faster than the numerator, such directional reversals may occur.

This type of reversal accompanying a changing lipid pool has been observed in other cases, with gestational lipemia providing a clear illustration. The normal elevation of total circulating lipids relative to the non-pregnant state results in decreased docosahexaenoic acid (DHA) as a percent of total lipids, while experiencing an increase in absolute DHA concentration (mg/dL plasma) [22]. To understand the dynamics in that case requires considering the total lipid pool size (total FA) in addition to DHA measured as both % total and a concentration. Using DHA only expressed as % total would lead one to conclude that DHA levels decrease during pregnancy, despite increasing in absolute concentration. This type of reversal was also observed in Bradbury et al. [23], who found that the relationship between LA and cholesterol reverses sign depending on expression format.

These previous findings raise the question of whether the common practice of only considering FA as % total results in an incomplete picture of many systems. Recent studies continue to use circulating FA biomarkers to address major cardiometabolic questions, but most of this evidence remains based on relative FA measures. Therefore, the field does not know how often conclusions would differ if the same FAs were analyzed as absolute concentrations while also considering total FA pool size. While prior work demonstrated that associations between FAs and lipid biomarkers can differ when FAs are expressed as % total versus concentrations [21], this issue has not been systematically evaluated in a large, nationally representative population with broad clinical phenotyping. In particular, it remains unclear whether expression-dependent reversals are limited primarily to lipid outcomes, or whether they also occur for non-lipid cardiometabolic biomarkers such as adiposity, blood pressure, glucose, and insulin. The 2011–2012 National Health and Nutrition Examination Survey (NHANES) provided a unique opportunity to address this gap because serum FAs were quantified by gas chromatography mass spectrometry and released as absolute concentrations, allowing direct comparison of FA-biomarker associations with each FA expressed as either % total or concentration. The current paper evaluated whether the direction and strength of associations between circulating FAs and cardiometabolic biomarkers differ by FA expression format, and whether these differences are related to variation in total FA pool size. These analyses show that the direction and strength of associations between circulating FAs and health outcomes often differ depending on the expression format and relationships with total FA. Caution is warranted when interpreting studies based on % total FA data alone.

## 2. Materials and Methods

Demographic, laboratory, and FA data from the 2011–2012 cycle were downloaded from the NHANES website (https://wwwn.cdc.gov/nchs/nhanes/continuousnhanes/default.aspx?BeginYear=2011, accessed 8 January 2026). Following the data flowchart shown in Supplementary Figure S1, data were analyzed from 2377 subjects (48.8% men, mean age 46.5 years; additional demographic information in Supplementary Table S1). Briefly summarizing the NHANES protocol, participants were randomly selected among those in the fasting sub-sample (fasting at their morning examination) and provided a 0.5 mL blood sample. FAs were measured using the modified methods of Lagerstedt et al. at the Centers for Disease Control and Prevention or their collaborators/subcontractors’ laboratories [24,25]. Following alkaline hydrolysis, hexane extraction, and derivatization with pentafluorobenzyl bromide, FA esters were analyzed. Total FAs were hexane-extracted from the matrix (100 uL serum or plasma), and an internal standard solution was applied to monitor FA recovery.

Following the extract’s conversion to pentafluorobenzyl esters, the reaction mixture was injected into a capillary gas chromatograph column to separate individual FAs. Within 34 min, 30 individual FAs were detected with electron capture negative-ion mass spectrometry (Agilent GC model #7890A; MS model #5975C) and MSD Chemstation software (model: E.02.0049) by comparing peak analyte area of unknown to known in calibrator solution. Absolute concentrations (mmol/L) were determined by selected ion monitoring using ratios of stable-isotope-labeled deuterated internal standards. These data were then used to generate compositional data (% total) for each FA.

All statistical analyses were conducted in R (version 4.4.0) and RStudio (version 2023.06.1+524) following the guidelines provided by the NHANES website [26,27]. The survey (version 4.4-2) package was used to account for the NHANES sampling strategy as recommended [28]. Common lipid and non-lipid outcome measures were considered and extracted. The distributions of outcome variables were inspected and log-transformed where appropriate; here, that was done for triglycerides and insulin. Association plots in this manuscript show Pearson’s correlation coefficients and *p*-values, both calculated accounting for the survey design. Regression models (svyglm) were also investigated for the sign reversal phenomenon in the regression coefficients and changes in the significance of *p*-values. A basic model was adjusted for only age and sex, while a more complex model was adjusted for age, sex, race, blood pressure medication, diabetes medication, cholesterol medication, food intake total calories, food intake n6-to-n3 ratio, and alcohol intake. These covariates were not from any specific interest—they were selected to build a model comparable to what is typically found in the literature in order to evaluate the extent of the sign and significance reversal. *p*-values were not corrected, as the goal was to show the reversal phenomenon, not to accurately assess all the possible associations.

## 3. Results

### 3.1. Associations of Linoleic Acid and Oleic Acid (Expressed as Percentage of Total Fatty Acids or Absolute Concentrations) with Common Clinical Biomarkers

**Figure 1:**
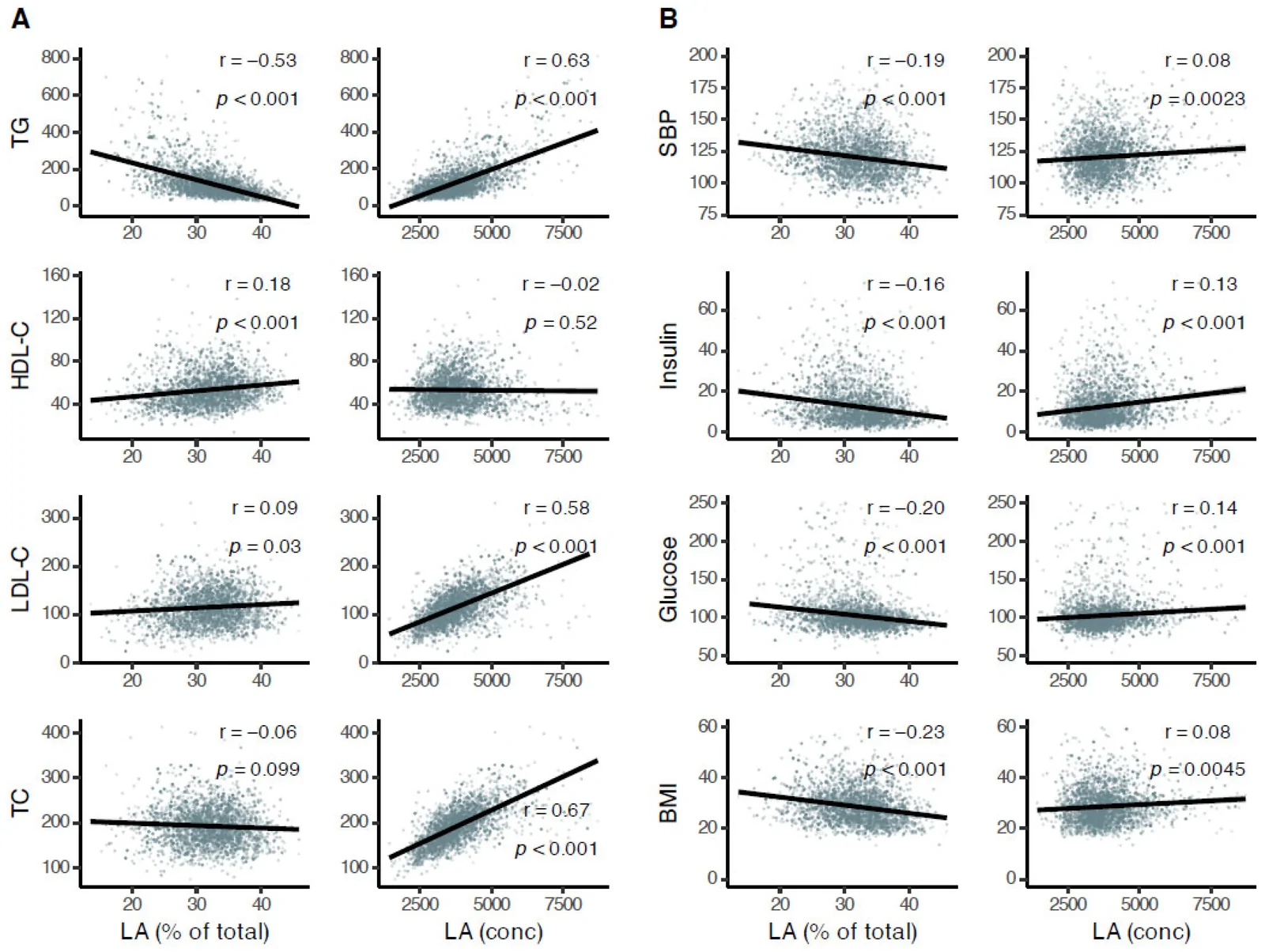
Associations between linoleic acid (LA) and four lipid-based (**A**) and four non-lipid (**B**) clinical biomarkers. LA is expressed as both % total and concentration (mol/L), and many of the associations change direction when switching from one method of expression to the other. Additional information, including the coefficients and *p*-values, is in Supplementary Table S2. Abbrev. TG = triglycerides; HDL-C, high-density cholesterol; LDL-C, low-density cholesterol; TC = total cholesterol; SBP = systolic blood pressure; BMI = body mass index.

Figure 1 shows the associations of LA, expressed as % total or absolute concentrations, with several lipid and non-lipid clinical biomarkers. The directions of the associations reverse depending on how LA is expressed. LA’s association with circulating triglycerides (TG) shows a marked reversal, while more subtle reversals are seen with HDL-C and total cholesterol (TC) (Figure 1A). Since LA is itself a FA that primarily resides in complex lipids such as TG, cholesterol esters and phospholipids, it was also important to examine associations with non-lipid clinical biomarkers (Figure 1B). Associations between LA and systolic blood pressure, BMI, insulin and glucose levels all showed reversals when LA was expressed as a % total versus absolute concentrations. Specifically, the associations flipped from negative to positive when LA was expressed as absolute concentration.

In contrast, for oleic acid (OA; 18:1, n9), the associations were mostly consistent in direction when the data were expressed as % total or absolute concentrations. Supplementary Figure S2 shows the relationships between OA and the same biomarkers examined in Figure 1. While the directions of the associations were more consistent with this FA, there were still reversals in direction with LDL-C and TC.

**Figure 2:**
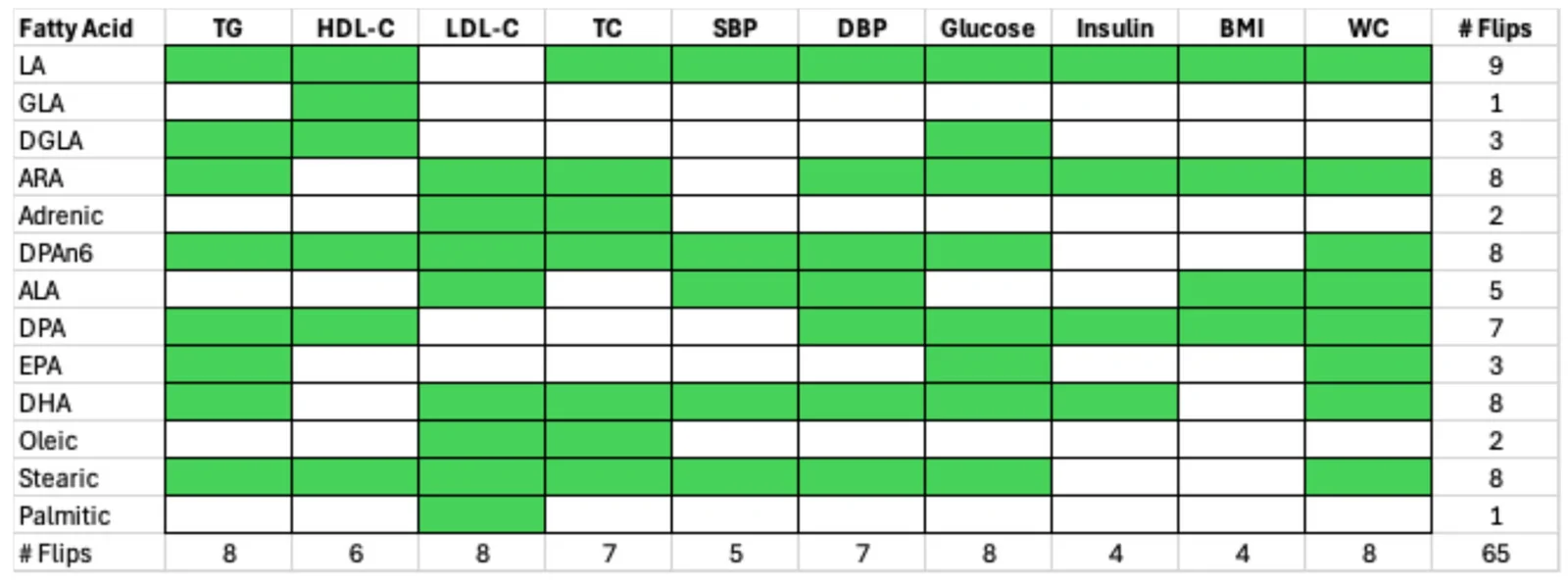
This image shows in green cells which correlations between individual fatty acids (rows) and clinical biomarkers (columns) reverse direction or “flip” depending on whether the FA is expressed as % total or as absolute concentration. The numerical results, including the correlation coefficients and *p*-values, are in Supplementary Table S2. Abbrev. # = number; TG = triglycerides; LDL-C = low density cholesterol; HDL-C = high-density cholesterol; TC = total cholesterol; SBP = systolic blood pressure; DBP = diastolic blood pressure; BMI = body mass index; GLA = γ-linolenic acid; DGLA = dihomo-γ-linolenic; DPA = docosapentaenoic acid; ALA = α-linolenic acid.

Overall, many associations reversed direction based on how the FA was expressed. Figure 2 shows which correlations reverse direction when the data are expressed as % total versus absolute concentration for 13 FAs and 10 clinical biomarkers. The most relationship reversals were observed with LA, with nine, and ARA, docosapentaenoic acid (DPA; 22:5, *n*-6), DHA, and stearic acid (SA, 18:0), all with eight. In many cases, the direction and magnitude of the correlation coefficient markedly changed. Additionally, the statistical significance sometimes also shifted, such that the correlation with the FA expressed as concentration was statistically significant, while its % total coefficient was not, or vice versa. Further, this was not limited to correlations—these reversals in both the association direction and statistical significance also occur in more complex regression models. Supplementary Tables S3 and S4 show this phenomenon for regression models including more covariates and significance. While the addition of covariates can alter estimates or the reversal phenomenon in specific cases, it does not make the reversal phenomenon disappear. Both models include the same covariates, which are held constant when estimating the FA effects. The reversal is connected to the mathematical relationship between FA % total and FA concentration, and how those vary with total FA.

### 3.2. Comparing % Total and Concentrations of FAs in the Context of Total Circulating FAs

**Figure 3:**
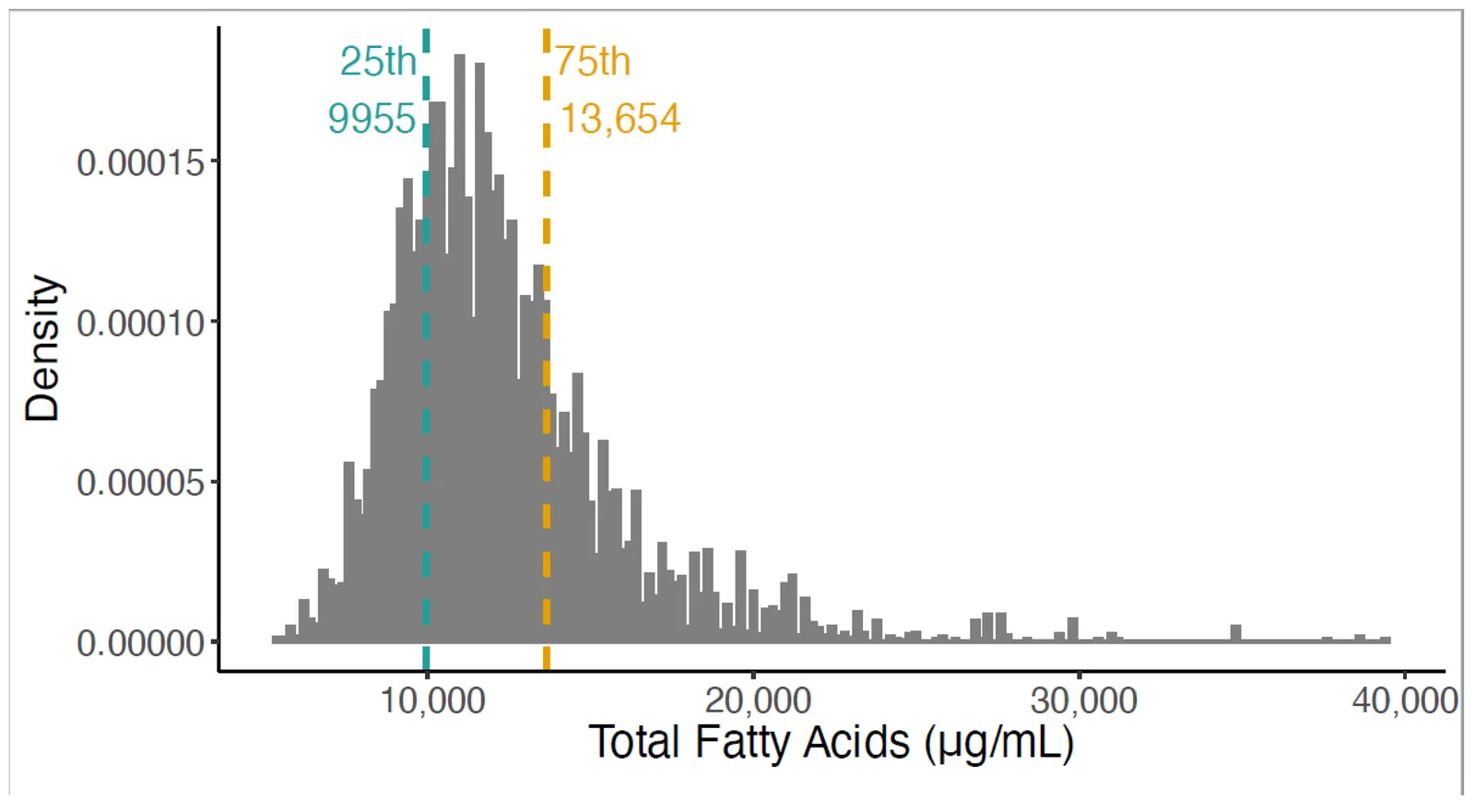
Distribution of total fatty acids; the 25th and 75th percentile lines indicate the first (Q1) and fourth (Q4) quartiles.

These findings are similar to the findings on ARA and DHA in pregnant women, where large increases in total FA were reducing the relative percentages of those individual FAs. To better understand how the composition of the NHANES FA dataset varied with total circulating FA, FA profiles were expressed both ways for individuals in the first (q1) and fourth (q4) quartiles of total FA. The distribution of total FA is shown in Figure 3. The quantile-based approach using % total data has been widely applied in epidemiologic studies to examine associations between circulating FAs and clinical biomarkers or endpoints [1,12,13,14,16,17,18,20].

**Figure 4:**
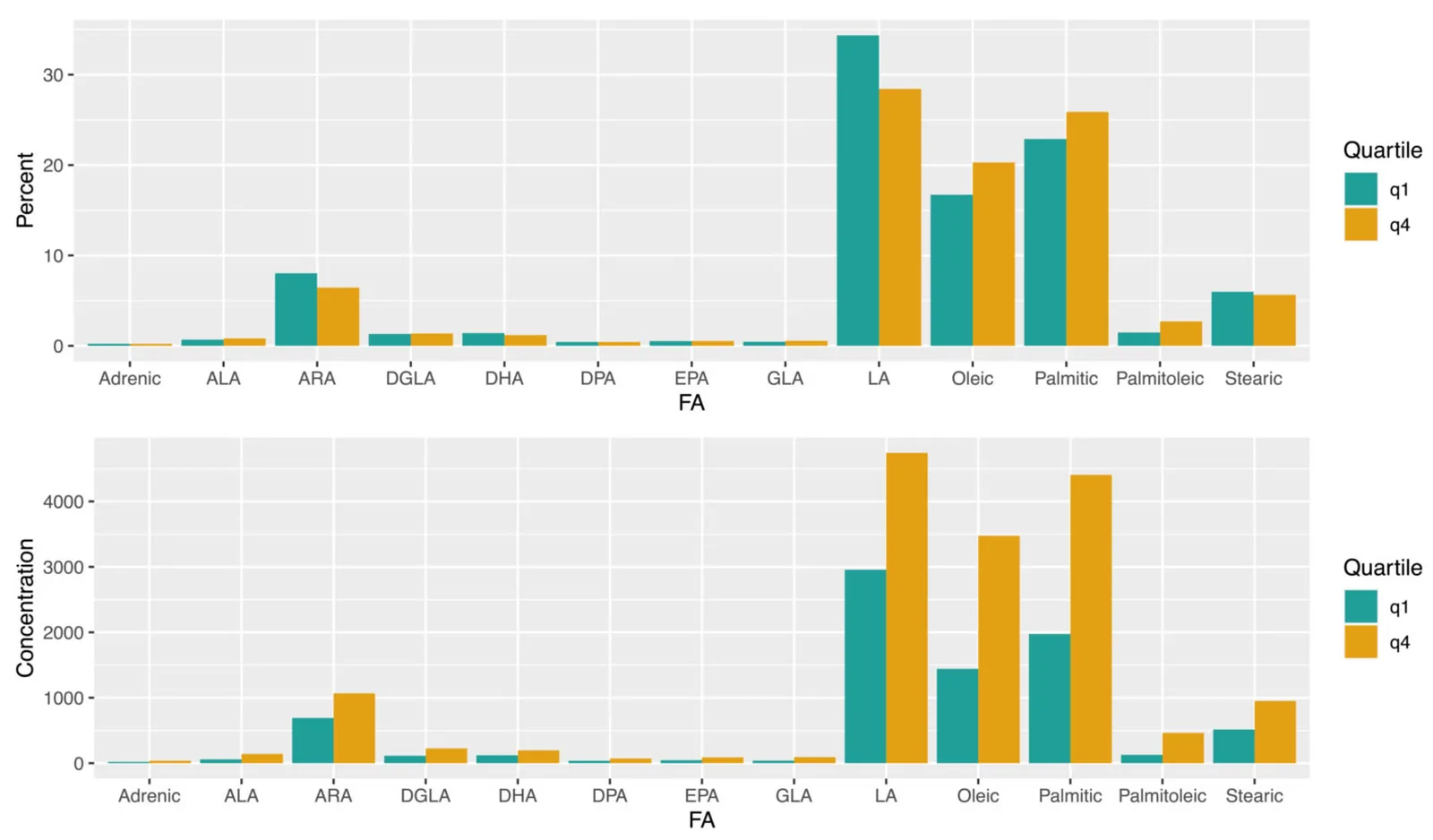
FA quantities expressed as % total (**Top**) or absolute concentrations (**Bottom**) in the first (q1) and fourth (q4) quartiles of total plasma FA levels as shown in Figure 3.

Figure 4 illustrates that the overall average FA composition differs markedly between q1 and q4, whether expressed as % total or as absolute concentrations. In the lower panel, as expected, the concentrations of all FAs were higher in q4 than q1, with notable increases in the absolute concentrations of multiple FAs, including LA, ARA, OA, palmitic, and stearic acid. When this was compared with FAs expressed as % total, the percentages of some FAs, like OA and palmitic acid, also increased with total FA from q1 to q4, though the magnitude was reduced compared with concentration. Importantly, although LA and ARA showed a 1.6-fold and 1.7-fold increase in absolute concentrations, respectively, their % total values decreased by approximately 15%, highlighting how misleading % total data can be in the context of rising total FA levels.

**Figure 5:**
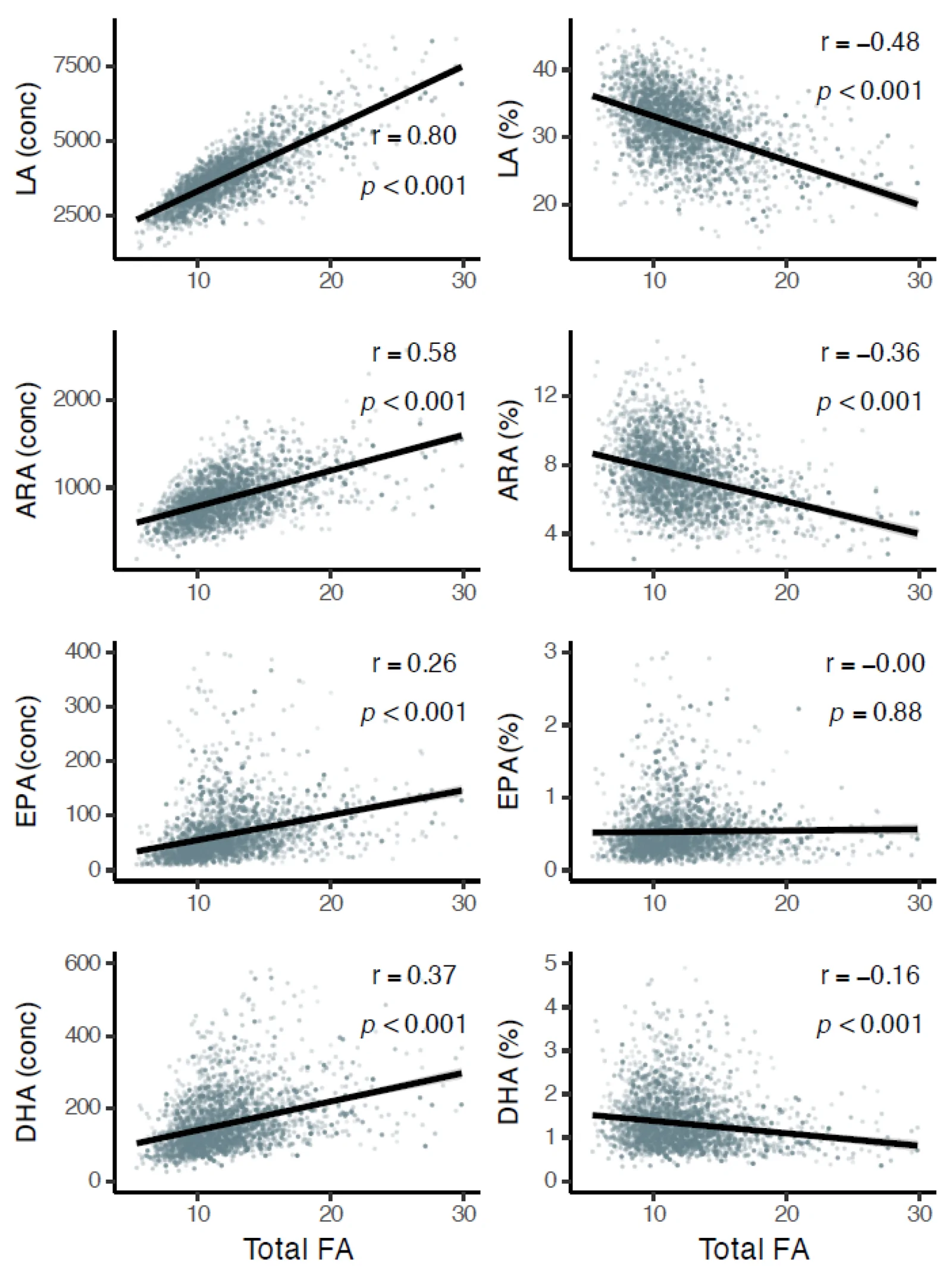
Plots of four individual FAs [expressed as both concentration (μmol/L) and % total] plotted as a function of total FA concentration (mmol/L).

Figure 5 further illustrates this phenomenon by showing relationships between total FA concentration and four important FAs (LA, ARA, EPA, and DHA) expressed both as % total and as concentrations. For three of these (LA, ARA, and DHA), the direction of association with total FA reverses when switching from % total to concentration. That is, as total FA levels rise, these FAs decline in % total terms but increase in concentration. In contrast, EPA demonstrates a positive association with total FA levels in both measures, although the strength of association is considerably greater when using concentrations compared to % total.

### 3.3. Associations of Total Fatty Acids with Common Clinical Biomarkers

**Figure 6:**
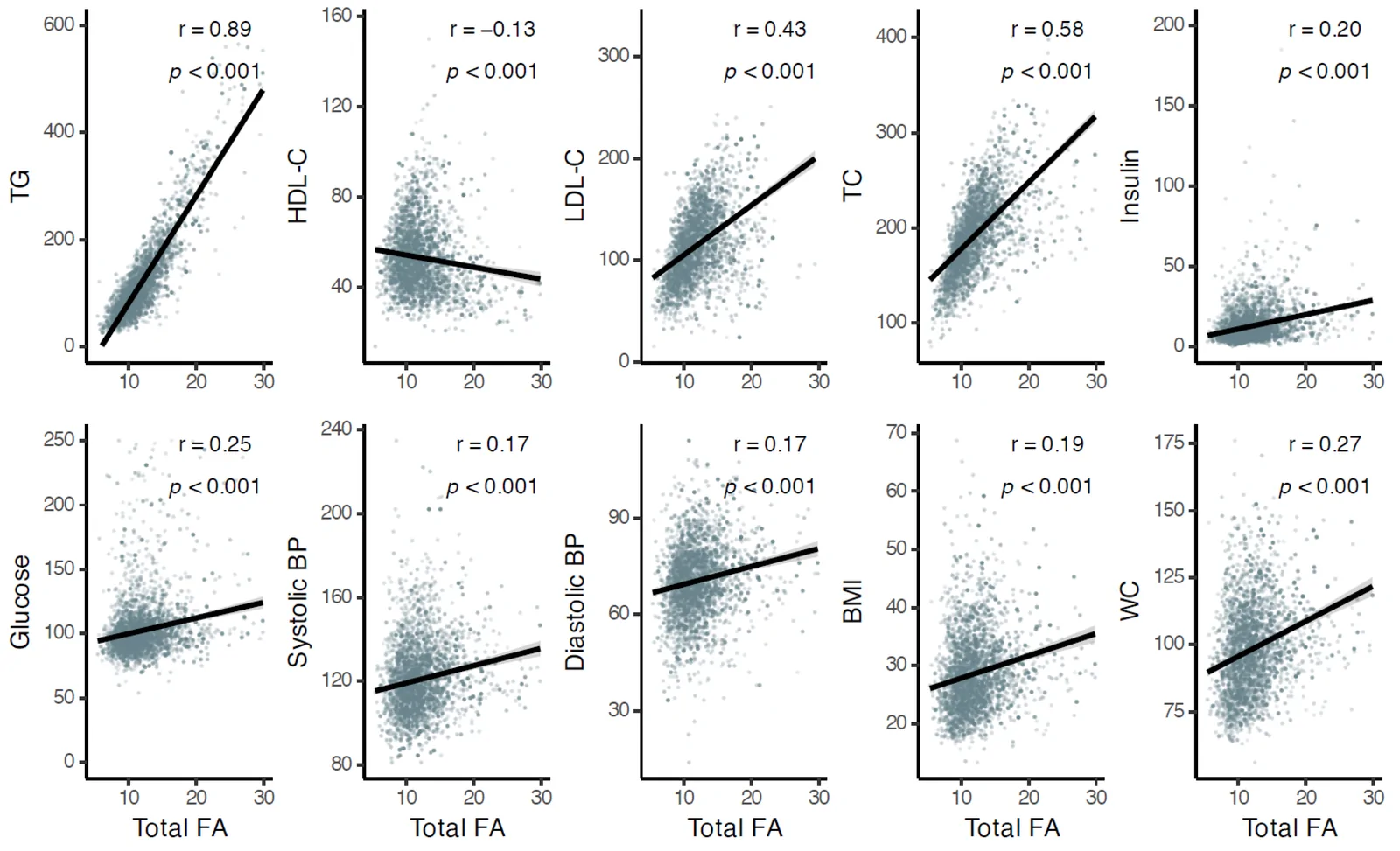
Clinical biomarkers plotted as a function of total FA concentrations. Abbreviations include: TG, triglycerides; HDL-C, high-density cholesterol; LDL-C, low-density cholesterol; TC, total cholesterol; BP, blood pressure; BMI, body mass index; WC, waist circumference.

Figure 6 illustrates the significant associations between total FA and ten clinical biomarkers. Nine (TG, LDL-C, TC, insulin, glucose, systolic and diastolic blood pressure (BP), BMI, and waist circumference) were positively associated with total FA, while one, HDL-C, was negatively associated with total FA. This highlights the importance of total FA in these types of analyses and points to why these reversals appear. For example, suppose both total FA and LA concentration increase with outcome Y and LA concentration is positively associated with Y. If total FA increases more rapidly than LA concentration, then LA will comprise a smaller proportion of the total FA pool, possibly producing a negative association between LA % total and Y.

## 4. Discussion

The 2011–2012 NHANES, with its quantitative mass spectrometry data, extensive clinical biomarker panel, and large cohort, provided the opportunity to compare associations between individual FAs and various cardiometabolic biomarkers with FAs expressed either as % total or as absolute concentrations. While our previous work [21] has shown both mathematically and empirically that reversals can occur, this paper highlights the potential for such reversals in a large, commonly used epidemiological cohort. Notably, there was at least one reversal in the direction of an association when switching between formats for the 13 most common FAs, including LA, ARA, DPA, DHA, and SA. A particularly striking example was the reversal in the association between LA, the primary dietary 18-carbon *n*-6 PUFA, and nine lipid and non-lipid biomarkers. Further, this was also observed in more complex statistical regression models with covariates more typically used in epidemiological analyses.

These reversals arise from the mathematical relationship between FA concentration, total FA concentration, and FA % total. Specifically, for a given FA, that FA % total equals the FA concentration divided by total FA. Therefore, FA % total is not simply an alternative scale for FA concentration; they are different quantities representing different information. FA % total reflects the relative level of a FA in the total FA pool, but changes may be due to either the numerator or denominator. If total FA increases faster than the concentration of an individual FA, that FA may decline as % total even while its absolute concentration increases. This is exactly what was observed for multiple FAs and biomarkers in NHANES in the current study. Consequently, FA concentration and total FA then provide complementary information. Importantly, these reversals were not explained by differences in covariate adjustment, as the % total and concentration models used the same covariates, and the phenomenon persists under alternative covariate specifications.

Another example of this sign reversal phenomenon was described by Bradbury et al. [23]. Their data showed a reversal in the direction of the association between LA and cholesterol depending on which method (% total or concentration) was used to express the FA data. They claimed FA % was a better predictor of cholesterol; however, in this case, “predictor” did not refer to statistical prediction, but meant that the observed relationship was in the “expected” direction. They also noted that total FA played a role. The role of total FA was noted in a different context by Mocking et al. [29], who looked at the correlations between FAs expressed both ways and found the result depended on the correlation with total FA, but they did not investigate associations with biomarker outcomes.

Because FA representation can alter the direction of observed associations, these findings have broad implications for studies relating circulating FAs to clinical biomarkers and potentially major clinical outcomes, including cardiovascular events and all-cause mortality. These results are not unique to NHANES or the other two cohorts [21], as the mathematical relationships are the same regardless of cohort, sampling methods, or study design. Much of the epidemiologic literature reports FAs as % total, implicitly treating relative composition as though it reflects the independent abundance of individual FAs [1,2]. However, when total FA pool size varies across individuals or across levels of a clinical biomarker or outcomes, % total may conflate changes in the FA of interest with changes in the denominator. Absolute concentrations can therefore provide additional, and often directionally different, information from % total values. Interestingly, this study shows the total FA concentration itself can be the strongest predictor of clinical biomarker levels, yet total FA is rarely reported or incorporated into epidemiologic analyses because it is not typically quantified in standard GC-FID-based datasets. These results suggest that total FA pool size may be an important but underappreciated feature of circulating FA biology.

This phenomenon may also help explain major discrepancies in the scientific literature. For example, the mixed results for associations between various FAs and coronary heart disease (CHD) mortality. Consistent with previous findings, a recent meta-analysis by Shi et al. [2], which included 49 non-overlapping studies analyzing circulating FAs expressed as % total, reported significant inverse associations of *n*-3 HUFAs and LA with both CHD and stroke mortality. However, supplementary NMR quantification of FAs (their Supplementary Table S3) from the same study, which examined 139,538 individuals from the UK Biobank and INTERVAL cohorts, showed reversals in hazard (HR) ratios for LA and CHD depending on whether FAs were expressed as relative percentages or absolute concentrations [2]. Additionally, a recent study by Zhang et al. [3] reports strong positive associations between the circulating *n*-6/*n*-3 PUFA and HUFA ratios and the risk of all-cause, cancer-related, and cardiovascular mortality. GC-FID can also be used to quantify FAs using an odd-chain FA as an internal standard. In fact, it is the industry-standard method recommended by organizations like AOAC and the European Committee for Standardization (EN) for lipid analysis and biodiesel profiling. To calculate the concentration of an individual FA in a sample, a ratio of the FA of interest to an odd-chain FA is obtained, and a concentration is calculated using a standard curve. Because the ratio of ester-carbon to chain-carbon differs slightly between a target FA and the odd-chain internal standard, a Relative Response Factor is then applied to correct the calculation. This is the methodology utilized in our previous FA concentration versus % total analysis [21]. A key limitation of observational research like NHANES is that it cannot establish the same kind of causal relationships as randomized controlled trials (RCTs). Rather than manipulating FA intake, observational studies compare individuals who differ across many variables simultaneously. In NHANES, increasing LA % total corresponds with decreasing total FA levels, making it difficult to isolate the effect of LA alone using % total data. RCTs and prospective cohort studies can help address this, but they come with practical challenges. For example, LA is already consumed at high levels (~17 g/day) in the standard Western diet, making meaningful dietary reduction difficult to achieve and sustain in a trial setting. Suitable replacement oils are limited, and participant compliance is difficult. Importantly, when LA intake has been manipulated in an RCT, the results look quite different from what observational data might suggest. The Sydney Diet Heart Study, one of the few trials to actually increase LA intake, found it was associated with a marked rise in all-cause mortality and coronary deaths [30,31]. This divergence between observational findings and RCT outcomes is a critical point as the two types of evidence are not interchangeable, and conclusions drawn from one should not be assumed to hold in the other. 

## 5. Conclusions

Taken together, these findings show that the format used to express circulating FAs (% total, absolute concentrations, ratios, or total FA) can fundamentally shape the associations observed with clinical biomarkers. The frequent reversals documented here indicate that reliance on % total alone may provide an incomplete or potentially misleading view of FA-biomarker relationships, particularly when total FA pool size varies. In addition to the best practices suggested by Brenna and colleagues [32], future FA studies should, whenever possible, report absolute concentrations alongside relative percentages, explicitly evaluate total FA concentrations, and distinguish associations involving FA composition from those involving FA abundance. Until these measurement issues are more consistently addressed, caution is warranted when translating epidemiologic associations based primarily on % total FA data into biological interpretation, dietary guidance, or clinical recommendations.

## Data Availability

Data described in the manuscript are publicly and freely available without restriction at https://wwwn.cdc.gov/nchs/nhanes/continuousnhanes/default.aspx?BeginYear=2011, accessed 8 January 2026.

## Supplementary Materials

The following supporting information can be downloaded at: https://www.mdpi.com/article/10.3390/nu18172905/s1. Figure S1. Data flowchart showing the path to our final sample size of n = 2377 adults. It is worth noting that the reversal phenomena in this paper does not depend on these choices, they are easily observable in subsets with alternative criteria. Figure S2. Associations between oleic acid (OA) and four lipid-based (A) and four non-lipid (B) clinical biomarkers. OA is expressed as both % total and concentration (μmol/L) and unlike Figure 1 most of the associations do not change direction when switching from one method of expression to the other. Additional information including the coefficients and *p*-values are in Supplementary Table S2. Abbrev. TG = triglycerides; HDL-C, high-density cholesterol; LDL-C, low density cholesterol; TC = total cholesterol; SBP = systolic blood pressure; BMI = body mass index. Table S1. Characteristics of the NHANES sample used in this manuscript. Table S2. Pearson correlation coefficients and *p*-values for fatty acids expressed as % total or concentration and various biomarkers. The table with green below indicates whether a sign reversal (aka “flip”) occurred and whether the significance changed (SigChange=yes) between expression formats. Table S3. Fatty acid beta coefficients and *p*-values from simple linear regression models are shown for both % total and concentration. The table with green below indicates whether a sign reversal (aka “flip”) occurred and whether the significance changed (SigChange=yes) between expression formats. Table S4. Fatty acid beta coefficients and *p*-values from more complex linear regression models are shown for both % total and concentration. The table with green below indicates whether a sign reversal (aka “flip”) occurred and whether the significance changed (SigChange=yes) between expression formats.

## Abbreviations

The following abbreviations are used in this manuscript:

FA	Fatty acid
PUFA	Polyunsaturated fatty acid
HUFA	Highly unsaturated fatty acid, >20C
LA	Linoleic acid
ARA	Arachidonic acid
EPA	Eicosapentanoic acid
DHA	Docosahexanoic acid
OA	Oleic acid
SA	Stearic acid
