# Supplementary material for: The Influence of Fatty Acid Expression Methods on Associations with Cardiometabolic Biomarkers: Evidence from NHANES 2011–2012
Source: Nutrients. 2026 Sep 4;18(17):2905. doi: 10.3390/nu18172905 (PMC13566945; doi:10.3390/nu18172905)
Supplement: Supplementary file 1 [file nutrients-18-02905-s001.zip › Figure S1 and Figure S2.pdf]

Supplementary Material

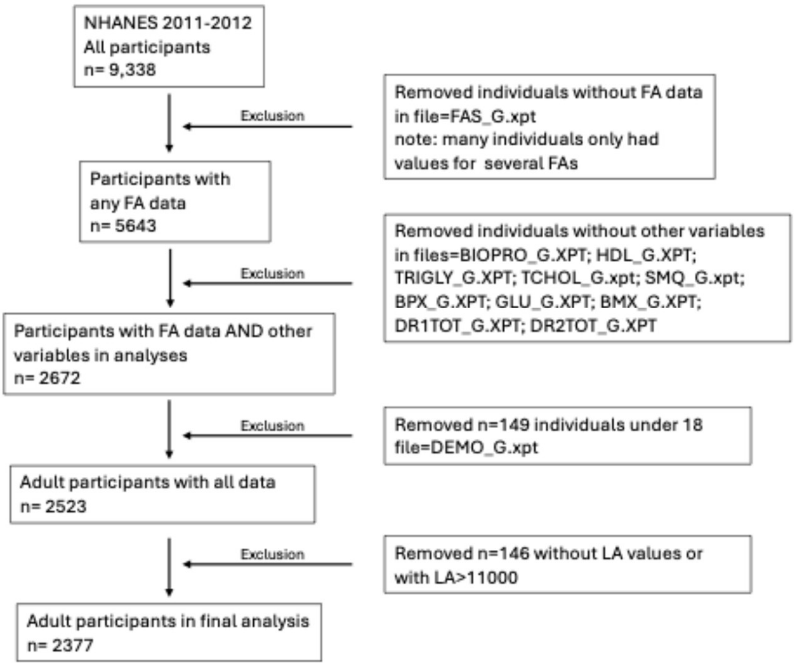

Supplementary Figure S1. Data flowchart showing the path to our final sample size of n=2377 adults. It is worth noting that the reversal phenomena in this paper does not depend on these choices, they are easily observable in subsets with alternative criteria.

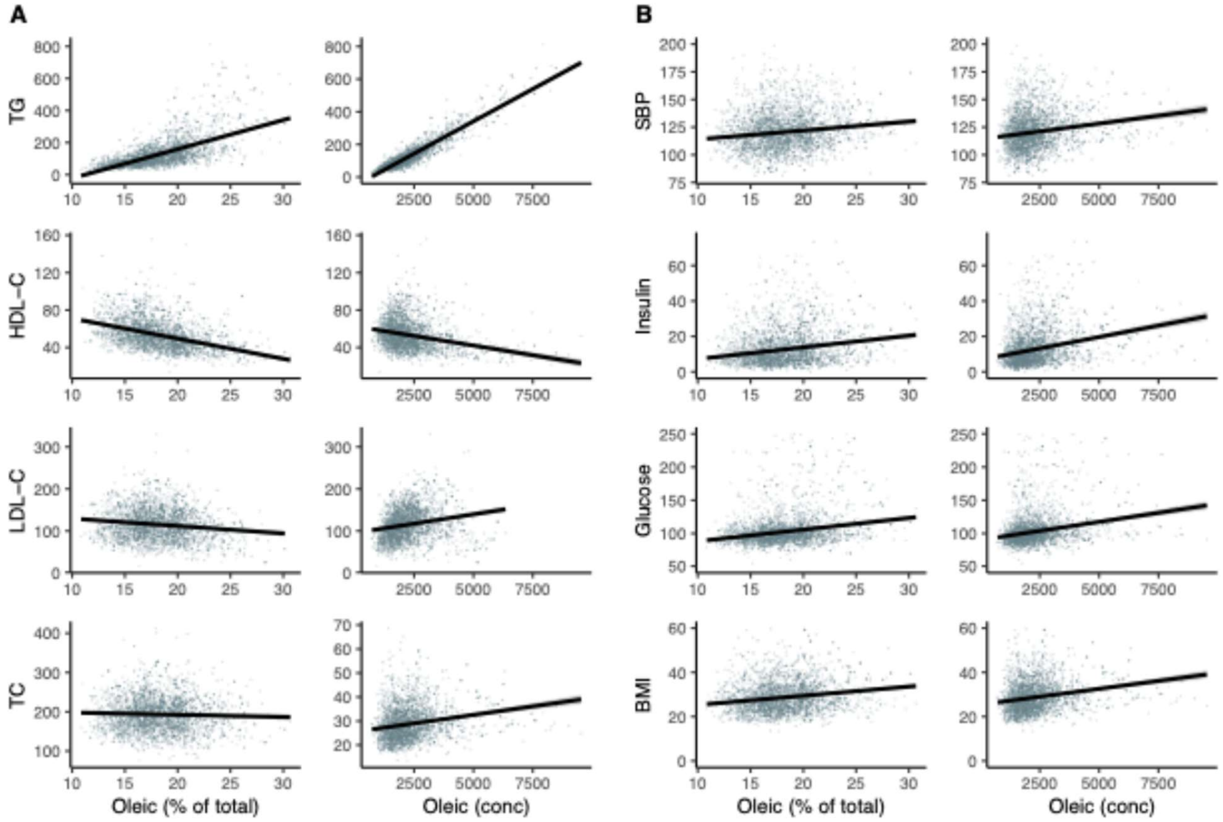

Supplementary Figure S2. Associations between oleic acid (OA) and four lipid-based **(A)** and four non-lipid **(B)** clinical biomarkers. OA is expressed as both % total and concentration (µmol/L) and unlike Figure 1 most of the associations do not change direction when switching from one method of expression to the other. Additional information including the coefficients and p-values are in Supplementary Table S2. Abbrev. TG = triglycerides; HDL-C, high-density cholesterol; LDL-C, low density cholesterol; TC = total cholesterol; SBP = systolic blood pressure; BMI = body mass index.
